# Supplementary material for: Cognitive functioning in context: Leisure activity engagement, social capital, and urbanicity-rurality interplay
Source: PLoS One. 2026 Jan 21;21(1):e0339496. doi: 10.1371/journal.pone.0339496 (PMC12822974; doi:10.1371/journal.pone.0339496)
Supplement: S1 File — Supplemental Materials for Cognitive Functioning in Context: Leisure Activity Engagement, Social Capital, and Urbanicity-Rurality Interplay Manuscript. (DOCX) [file pone.0339496.s001.docx]

**Supplemental Materials for** **Cognitive Functioning in Context: Leisure Activity Engagement, Social Capital, and Urbanicity-Rurality Interplay Manuscript**

**VIQ**

**Cognitive Engagement and Social Capital.** All cognitive engagement measures were modestly associated with VIQ after accounting for covariates (See S12 and S13 Tables, Model 1). Specifically, individuals that engaged in 15 minutes per day on hobbies scored about a fifth of standard deviation higher in VIQ compared to those that did not report participating in any hobbies (Cohen’s *d*=0.20)^[[1]](#footnote-1)^. IQ effect sizes were calculated according to the population expected standard deviation of 15 rather than the study sample of 11.9. Better verbal IQ was associated with greater cognitive demand and number of hobbies. For example, individuals that participated in 2 hobbies that were on average moderately demanding scored about a fourth of standard deviation higher in VIQ compared to those that only report 1 hobby that was scored a 2 or had some cognitive demand (Cohen’s *d*=.25)^[[2]](#footnote-2)^. Analyses with qualitative measures were restricted to those that reported their specific hobbies, thus minimum reports represent those with at least 1 hobby that was at least somewhat cognitively demanding. Social capital was not associated with VIQ after accounting for covariates and cognitive engagement.

**IRR and Engagement-IRR Moderation.** Parameter estimates and model fit statistics for IRR and the interaction with cognitive engagement on VIQ are reported in S12 and S13 Tables under Models 2 and 3, respectively. Across models, cognitive engagement was generally consistent for both quantitative (B_HPW_>=2.91, *p*<0.001) and qualitative (B_Demand_>=5.33, *p*<0.001; B_Number_>=4.06, *p*<0.001) measures with the inclusion of IRR or the moderation by IRR. IRR was not associated with VIQ after accounting for hours of engagement (B_IRR_=-4.52, *p*=0.05) or cognitive demand (B_IRR_=-5.52, *p*=0.06). We did not evidence moderation of IRR with HPW (B=-3.44, *p*=0.28) or cognitive demand (B=4.37 *p*=0.70). Adding IRR or the interaction with HPW or cognitive demand did not improve fit $(\Delta$χ2 (1)>=3.7, *p>*=0.05) for any model.

**SCI and IRR Moderation.** Model 4 retained IRR and included the interaction of SCI by IRR (see S12 and S13 Tables). Associations of SCI on VIQ did not vary by IRR for hours of engagement (B=-0.22, *p*=0.21; see S1 Figure 1a) and cognitive demand (B=-0.35, *p*=0.15; see S1 Figure 1b). Associations of cognitive engagement remained consistent with the addition of SCI moderated by IRR. Of note, the association of SCI (B=0.08, *p*=0.02) slightly increased and reached significance with the inclusion of SCI-IRR moderation. For VIQ, the best model based on lowest AIC and parsimony was Model 2 or including IRR for quantitative and qualitive activity engagement. For completeness, we illustrate the interaction between SCI and IRR on VIQ performance across hours of engagement (S1 Figure 1b) and cognitive demand (S1 Figure 1b).

**PIQ**

**Cognitive Engagement and Social Capital.** All cognitive engagement measures were modestly associated with PIQ after accounting for covariates (See S14 and S15 Tables , Model 1). Specifically, individuals that engaged in 15 minutes per day on hobbies scored about a fifth of standard deviation higher in PIQ compared to those that did not report participating in any hobbies (Cohen’s *d*=0.19)^[[3]](#footnote-3)^. Better performance IQ was associated with greater cognitive demand and number of hobbies. For example, individuals that participated in 2 hobbies that were on average moderately demanding scored about a fourth of standard deviation higher in VIQ compared to those that only report 1 hobby that was scored a 2 or had some cognitive demand (Cohen’s *d*=.21)^[[4]](#footnote-4)^. Of note, number of cognitive hobbies was not related to PIQ (B_Number_=0.91, *p=*0.40), see S15 Table. Social capital was not associated with PIQ after accounting for covariates and cognitive engagement.

**IRR and Engagement-IRR Moderation.** Parameter estimates and model fit statistics for IRR and the interaction with cognitive engagement on PIQ are reported in S14 and S15 Tables under Models 2 and 3, respectively. Across models, cognitive engagement was generally consistent for both quantitative (B_HPW_>=2.76, *p*<0.001) and qualitative (B_Demand_>=6.82, *p*<0.001) measures with the inclusion of IRR or the moderation by IRR. IRR was not associated with PIQ after accounting for hours of engagement (B_IRR_=-2.78, *p*>=0.38) or cognitive demand (B_IRR_=-3.98, *p*=0.32). We did not evidence moderation of IRR with HPW (B=-3.35, *p*=0.45) or cognitive demand (B=29.15 *p*=0.07). IRR associations with PIQ did reach significance (B=-11.18 *p*=0.02) after including the interaction between cognitive demand and IRR, however including the moderation of IRR with cognitive engagement did not improve fit for either hors of engagement $(\Delta$χ2 (1)>=0.6, *p*=0.44) or cognitive demand $(\Delta$χ2 (1)=3.3, *p*=0.07).

**SCI and IRR Moderation.** Model 4 retained IRR and included the interaction of SCI by IRR (see S14 and S15 Tables). Associations of SCI on PIQ varied by IRR for hours of engagement (B=-0.65, *p*=0.006; see S2 Figure S2a) and cognitive demand (B=-0.95, *p*=0.004; see S2 Figure S2b). Associations of cognitive engagement remained consistent with the addition of SCI moderated by IRR. Models including SCI-IRR moderation showed a significant increase in model fit for quantitative and qualitive activity engagement $(\Delta$χ2 (1)=7.4, *p*>=0.01). We illustrate the interaction between SCI and IRR on PIQ performance across hours of engagement in S2 Figure 2b and S2 Figure 2b for cognitive demand.

| S1 Table. Cognitive activity engagement item descriptive statistics. | | | | | | | |
| --- | --- | --- | --- | --- | --- | --- | --- |
|  |  | *N* | *Mean* | *SD* | *Var.* | *Min* | *Max* |
| Sum Cognitive HPW | | 1189 | 1.50 | 0.72 | 0.52 | 0 | 3.2 |
|  | reading for fun? | 1189 | 1.91 | 1.91 | 3.66 | 0 | 8.0 |
|  | spending time on a hobby? | 1189 | 2.21 | 2.26 | 5.13 | 0 | 8.0 |
|  | playing a musical instrument? | 1142 | 0.58 | 1.46 | 2.12 | 0 | 8.0 |
| *Note*: N’s reflect those with IRR and IQ scores; HPW=Hours per week. | | | | | | | |

|  | Cognitive Demand Subsample  *M*  N=741 | Missing  Cognitive Demand  *M*  N=130 | t | DF | *p* |
| --- | --- | --- | --- | --- | --- |
| Age | 33.0 | 32.6 | 0.87 | 869 | 0.39 |
| Female | 0.51 | 0.5 | 0.8 | 869 | 0.42 |
| White | 93% | 92% | 0.39 | 869 | 0.70 |
| Hispanic | 6% | 4% | 0.85 | 869 | 0.40 |
| Education | 17.14 | 16.19 | 3.44 | 867 | <0.001 |
| Occupation | 6.04 | 5.63 | 2.77 | 859 | 0.006 |
| FSIQ | 112 | 107.6 | 4.1 | 869 | <0.001 |
| VIQ | 109 | 105.3 | 3.42 | 869 | <0.001 |
| PIQ | 114.1 | 109.1 | 4.05 | 869 | <0.001 |
| SCI | -0.48 | -0.43 | -0.72 | 869 | 0.47 |
| IRR | 0.35 | 0.36 | -0.57 | 869 | 0.57 |

S2Table. Descriptives for the cognitive demand subsample.

*Note.* Analytic sample N reflects those not missing on cognitive demand score. Missing refers to those in analytic sample that engaged in some time on hobbies but didn’t report and hobbies. FSIQ=Full Scale IQ; VIQ=Verbal IQ; PIQ=Performance IQ; Demand=Average cognitive hobby demand; Number= Number of cognitive hobbies.

S3 Table. Partial Correlation and Intraclass Correlation Coefficients (ICC), removing half-siblings and siblings living together.

|  | ICC^R^ | | | | **Model Fit** | **ICC Comparisons ∆χ2 (df) *p*** | | |
| --- | --- | --- | --- | --- | --- | --- | --- | --- |
|  | **MZ** | **DZ** | **Con** | **AD** | **2LL (K)** | **MZ = DZ** | **DZ = Con** | **Con = AD** |
| **HPW** | 0.35 | 0.04 | 0.11 | 0.01 | 2394.9 (15) | **7.4 (2)** | 0.5 (2) | 5.1 (2) |
| N=1118 |  |  |  |  |  | 0.02 | 0.78 | 0.08 |
| **Demand** | 0.33 | 0.12 | 0.00 | 0.00 | -111.0 (15) | 1.8 (2) | 0.8 (2) | 0.5 (2) |
| N=698 |  |  |  |  |  | 0.41 | 0.67 | 0.78 |
| **Number** | 0.00 | 0.22 | 0.00 | 0.00 | 653.7 (15) | 4.2 (2) | 3 (2) | 0 (2) |
| N=698 |  |  |  |  |  | 0.12 | 0.22 | 1.00 |
| **SCI** | 0.34 | 0.47 | 0.05 | 0.09 | 8333.8 (15) | 2.6 (2) | **17.1 (2)** | **21.7 (2)** |
| N=1130 |  |  |  |  |  | 0.27 | <0.001 | <0.001 |
| **IRR** | 0.31 | 0.32 | 0.31 | 0.16 | -1797.0 (15) | 0.7 (2) | 0.1 (2) | 3.3 (2) |
| N=1130 |  |  |  |  |  | 0.70 | 0.95 | 0.19 |

*Note*. N's reflect those with IRR (Index of Relative Rurality) and IQ scores. Correlations adjusted for sex, age, project, adopted status, race, ethnicity, and live together. ICC^R^=ICC excluding up to 25 half-siblings in control or additional biological siblings in adoptive families and up to 46 participants that live together; -2 LL= -2 log-likelihood; HPW=Log-transformed cognitive hours per week; Demand= Log-transformed average cognitive demand of reported hobbies; Number= Log-transformed number of cognitive hobbies; Sibling type: MZ = monozygotic twins; DZ = dizygotic twins; Con = individuals in nonadoptive (“control”) families; AD = individuals in adoptive families. Bolded = *p* < .05.

S4 Table. Pearson partial correlation coefficients among education, occupational complexity, cognitive engagement, social capital and rurality variables.

| **Table S4a (N=1221)** | **Variables** | **Educational Attainment** | **Occupational Complexity** | **SCI** | **IRR** | **HPW Cognitive (LN)** |
| --- | --- | --- | --- | --- | --- | --- |
|  | **Occupational Complexity** | **0.48** |  |  |  |  |
|  | **SCI** | 0.02 | 0.01 |  |  |  |
|  | **IRR** | **-0.25** | **-0.17** | **0.08** |  |  |
|  | **HPW Cognitive (LN)** | **0.07** | 0.03 | 0.05 | 0.01 |  |
| **Table S4b (N = 760)** | ***Variables*** | **Educational Attainment** | **Occupational Complexity** | **SCI** | **IRR** | **HPW Cognitive (LN)** |
|  | **Occupational Complexity** | **0.46** |  |  |  |  |
|  | **SCI** | 0.04 | 0.02 |  |  |  |
|  | **IRR** | **-0.24** | **-0.18** | **0.16** |  |  |
|  | **Average Cognitive Demand of Hobbies (LN)** | -0.01 | 0.03 | 0.01 | **-0.09** |  |
|  | **Number of Cognitive Hobbies (LN)** | 0.05 | 0.03 | 0.05 | 0.02 | **-0.08** |

*Note*. Correlations adjusted for sex, age, project, adopted status, race, ethnicity, and live together. Bolded = *p* < .05.

S5 Table. Parameter Estimates and Model Fit for individual Engagement on FSIQ, with and without socioeconomic adjustment

|  | HPW-  Unadj | | HPW-  Adj | | Demand- Unadj | | Demand-  Adj | | Number- Unadj | | Number-  Adj | |
| --- | --- | --- | --- | --- | --- | --- | --- | --- | --- | --- | --- | --- |
|  | **B** | **se** | **B** | **se** | **B** | **se** | **B** | **se** | **B** | **se** | **B** | **se** |
| **Fixed Effects** |  |  |  |  |  |  |  |  |  |  |  |  |
| Intercept | **105.88** | 1.72 | **107.73** | 1.59 | **107.94** | 2.22 | **110.29** | 2.06 | **110.01** | 2.18 | **112.45** | 2.03 |
| Female | **-1.63** | 0.72 | **-2.57** | 0.66 | -1.66 | 0.88 | **-2.72** | 0.82 | **-2.14** | 0.88 | **-3.18** | 0.82 |
| Age | 0.08 | 0.14 | -0.05 | 0.13 | -0.10 | 0.18 | -0.21 | 0.17 | -0.08 | 0.18 | -0.20 | 0.17 |
| Project | **4.06** | 1.51 | **3.88** | 1.38 | **6.14** | 1.87 | **6.05** | 1.75 | **5.90** | 1.88 | **5.84** | 1.75 |
| Adopted | **-4.49** | 1.11 | **-2.67** | 1.01 | **-4.36** | 1.44 | **-2.93** | 1.34 | **-4.29** | 1.44 | **-2.88** | 1.34 |
| Hispanic | **-3.21** | 1.89 | **-2.30** | 1.70 | -0.95 | 2.18 | -0.83 | 2.00 | -1.12 | 2.18 | -1.01 | 2.01 |
| White | 0.05 | 1.45 | -0.03 | 1.33 | 1.58 | 2.00 | 0.60 | 1.84 | 1.10 | 2.00 | 0.06 | 1.85 |
| Live Together | -3.25 | 2.17 | -2.08 | 1.90 | -2.39 | 2.64 | -1.38 | 2.37 | -2.89 | 2.64 | -1.87 | 2.37 |
| Education | - | - | **1.32** | 0.11 | - | - | **1.35** | 0.15 | - | - | **1.32** | 0.15 |
| Occupation | - | - | 0.37 | 0.19 | - | - | 0.16 | 0.26 | - | - | 0.22 | 0.27 |
| HPW | **3.09** | 0.39 | **3.06** | 0.37 | - | - | - | - | - | - | - | - |
| Demand | - | - | - | - | **6.65** | 1.63 | **6.99** | 1.54 | - | - | - | - |
| Number | - | - | - | - | - | - | - | - | **2.78** | 0.90 | **2.64** | 0.87 |
| **Random Effects** |  |  |  |  |  |  |  |  |  |  |  |  |
| σ^2^_BW_ AD | 13.66 | 12.25 | 2.36 | 9.28 | 0.00 | . | 0.00 | . | 4.72 | 20.80 | 0.00 | . |
| σ^2^_BW_ Con | 53.37 | 14.14 | 37.99 | 12.18 | 60.60 | 19.09 | 52.61 | 18.59 | 52.38 | 19.03 | 43.97 | 18.32 |
| σ^2^ _BW_ DZ | 56.43 | 10.34 | 38.74 | 8.74 | 44.82 | 12.50 | 36.00 | 10.95 | 42.72 | 12.67 | 34.10 | 11.10 |
| σ^2^ _BW_ MZ | 97.37 | 11.62 | 69.62 | 8.91 | 91.91 | 13.62 | 67.23 | 10.76 | 90.03 | 13.56 | 65.46 | 10.88 |
| σ^2^_WI_ AD | 123.61 | 15.84 | 109.61 | 13.46 | 147.01 | 17.36 | 122.52 | 14.49 | 144.35 | 26.69 | 125.39 | 14.83 |
| σ^2^ _WI_ Con | 73.44 | 10.87 | 70.13 | 10.35 | 67.13 | 14.83 | 62.98 | 14.68 | 73.11 | 15.96 | 67.94 | 15.54 |
| σ^2^ _WI_ DZ | 57.59 | 6.84 | 58.84 | 7.01 | 63.25 | 10.29 | 59.63 | 9.54 | 66.66 | 10.80 | 62.77 | 10.00 |
| σ^2^ _WI_ MZ | 22.10 | 2.59 | 22.26 | 2.65 | 26.87 | 4.63 | 26.44 | 4.55 | 27.90 | 4.82 | 29.02 | 5.02 |
| Model Fit |  | |  | |  | |  | |  | |  | |
| -2 LL | 8742 | | 8569.3 | | 5523 | | 5420.9 | | 5530 | | 5431.8 | |
| AIC | 8776 | | 8607.3 | | 5555 | | 5456.9 | | 5564 | | 5467.8 | |
| N Individual/Sibships | 1172/712 | | 1172/712 | | 733/537 | | 733/537 | | 733/537 | | 733/537 | |

*Note*. FSIQ=Full-Scale IQ; Unadj/Adj= Unadjusted/Adjusted for educational and occupational attainment; HPW=Log-transformed cognitive activity hours per week; Demand=Average cognitive hobby demand; Number= Number of cognitive hobbies; -2 LL= -2 log-likelihood; Random effects: σ2BW=between siblings and σ2WI=within siblings; subscript notes sibling type: AD=adoptive family siblings, Con=control family siblings, DZ=dizygotic twins, and MZ=monozygotic twins. Bolded parameters are significant *p* < .05

S6 Table. Model Parameters for SCI & IRR on FSIQ, with and without socioeconomic adjustment

|  | SCI-  Unadj | | | | SCI-  Adj | | | | IRR-  Unadj | | | | IRR-  Adj | | | |
| --- | --- | --- | --- | --- | --- | --- | --- | --- | --- | --- | --- | --- | --- | --- | --- | --- |
|  | **B** | | **se** | | **B** | | **se** | | **B** | | **se** | | **B** | | **se** | |
| **Fixed Effects** |  | |  | |  | |  | |  | |  | |  | |  | |
| Intercept | **110.30** | | 1.68 | | **112.07** | | 1.55 | | **110.29** | | 1.68 | | **112.03** | | 1.55 | |
| Female | **-1.99** | | 0.74 | | **-2.94** | | 0.67 | | **-2.00** | | 0.73 | | **-2.93** | | 0.67 | |
| Age | 0.05 | | 0.14 | | -0.08 | | 0.13 | | 0.06 | | 0.14 | | -0.07 | | 0.13 | |
| Project | **4.41** | | 1.55 | | **4.25** | | 1.41 | | **4.41** | | 1.54 | | **4.24** | | 1.41 | |
| Adopted | **-4.72** | | 1.16 | | **-2.91** | | 1.06 | | **-4.90** | | 1.15 | | **-3.01** | | 1.06 | |
| Hispanic | -3.24 | | 1.93 | | -2.21 | | 1.75 | | **-3.55** | | 1.92 | | -2.36 | | 1.75 | |
| White | 0.38 | | 1.49 | | 0.35 | | 1.37 | | 0.45 | | 1.49 | | 0.40 | | 1.37 | |
| Live Together | -2.62 | | 2.22 | | -1.45 | | 1.95 | | -2.38 | | 2.19 | | -1.35 | | 1.95 | |
| Education | - | | - | | **1.33** | | 0.11 | | - | | - | | **1.32** | | 0.12 | |
| Occupation | - | | - | | **0.39** | | 0.19 | | - | | - | | 0.37 | | 0.19 | |
| SCI | 0.04 | | 0.03 | | 0.04 | | 0.03 | | - | | - | | - | | - | |
| IRR | - | | - | | - | | - | | **-7.62** | | 2.58 | | -2.28 | | 2.50 | |
| **Random Effects** | |  | |  | |  | |  | |  | |  | |  | |  |
| σ^2^_BW_ AD | 20.47 | | 14.13 | | 4.46 | | 10.56 | | 21.93 | | 14.25 | | 5.00 | | 10.60 | |
| σ^2^_BW_ Con | 58.42 | | 15.18 | | 43.01 | | 13.19 | | 52.48 | | 14.97 | | 41.09 | | 13.12 | |
| σ^2^ _BW_ DZ | 60.77 | | 11.00 | | 41.66 | | 9.32 | | 58.41 | | 10.72 | | 42.32 | | 9.32 | |
| σ^2^ _BW_ MZ | 101.78 | | 12.00 | | 73.40 | | 9.23 | | 98.19 | | 11.78 | | 72.57 | | 9.19 | |
| σ^2^_WI_ AD | 132.71 | | 17.35 | | 119.79 | | 14.87 | | 131.40 | | 17.26 | | 119.79 | | 14.86 | |
| σ^2^ _WI_ Con | 73.48 | | 11.12 | | 69.08 | | 10.53 | | 76.16 | | 11.57 | | 70.39 | | 10.71 | |
| σ^2^ _WI_ DZ | 60.89 | | 7.22 | | 63.24 | | 7.49 | | 60.32 | | 7.13 | | 62.73 | | 7.43 | |
| σ^2^ _WI_ MZ | 21.63 | | 2.47 | | 21.97 | | 2.56 | | 22.77 | | 2.66 | | 22.26 | | 2.62 | |
| Model Fit |  | | | |  | | | |  | | | |  | | | |
| -2 LL | 8826.6 | | | | 8657.4 | | | | 8820.3 | | | | 8658.6 | | | |
| AIC | 8860.6 | | | | 8695.4 | | | | 8854.3 | | | | 8696.6 | | | |
| N Individual/Sibships | 1176/712 | | | | 1176/712 | | | | 1176/712 | | | | 1176/712 | | | |

*Note.* FSIQ=Full-Scale IQ; Unadj/Adj= Unadjusted/Adjusted for educational and occupational attainment; IRR=Index of Relative Rurality; SCI=Social Capital Index; -2 LL= -2 log-likelihood; Random effects: σ2BW=between siblings and σ2WI=within siblings; subscript notes sibling type: AD=adoptive family siblings, Con=control family siblings, DZ=dizygotic twins, and MZ=monozygotic twins. Bolded parameters are significant p < .05

S7 Table. Model Parameters for individual Engagement on VIQ, with and without socioeconomic adjustment

|  | HPW-  Unadj | | HPW-  Adj | | Demand- Unadj | | Demand-  Adj | | Number- Unadj | | Number-  Adj | |
| --- | --- | --- | --- | --- | --- | --- | --- | --- | --- | --- | --- | --- |
|  | **B** | **se** | **B** | **se** | **B** | **se** | **B** | **se** | **B** | **se** | **B** | **se** |
| **Fixed Effects** |  |  |  |  |  |  |  |  |  |  |  |  |
| Intercept | **104.06** | 1.67 | **105.96** | 1.52 | **106.25** | 2.20 | **108.84** | 2.01 | **107.46** | 2.14 | **110.21** | 1.96 |
| Female | **-2.51** | 0.69 | **-3.54** | 0.63 | **-2.74** | 0.87 | **-3.90** | 0.79 | **-3.16** | 0.86 | **-4.29** | 0.79 |
| Age | 0.10 | 0.13 | -0.02 | 0.12 | -0.10 | 0.17 | -0.21 | 0.16 | -0.06 | 0.17 | -0.17 | 0.16 |
| Project | 1.54 | 1.41 | 1.32 | 1.26 | 3.53 | 1.78 | **3.45** | 1.62 | 3.05 | 1.77 | 3.01 | 1.60 |
| Adopted | **-4.65** | 1.03 | **-2.72** | 0.92 | **-4.15** | 1.36 | **-2.63** | 1.24 | **-4.00** | 1.35 | **-2.53** | 1.23 |
| Hispanic | -3.11 | 1.89 | -2.25 | 1.70 | -1.20 | 2.22 | -1.15 | 2.01 | -0.98 | 2.18 | -1.07 | 1.99 |
| White | 0.72 | 1.40 | 0.73 | 1.27 | 2.36 | 1.99 | 1.26 | 1.80 | 2.15 | 1.97 | 0.98 | 1.79 |
| Live Together | -2.14 | 2.21 | -1.03 | 1.94 | -1.19 | 2.73 | -0.19 | 2.44 | -1.65 | 2.70 | -0.67 | 2.43 |
| Education | - | - | **1.39** | 0.11 | - | - | **1.51** | 0.14 | - | - | **1.46** | 0.14 |
| Occupation | - | - | 0.27 | 0.18 | - | - | 0.03 | 0.25 | - | - | 0.07 | 0.24 |
| HPW | **2.93** | 0.37 | **2.90** | 0.35 | - | - | - | - | - | - | - | - |
| Demand | - | - | - | - | **4.18** | 1.55 | **4.75** | 1.44 | - | - | - | - |
| Number | - | - | - | - | - | - | - | - | **4.06** | 0.83 | **3.83** | 0.78 |
| **Random Effects** |  |  |  |  |  |  |  |  |  |  |  |  |
| σ^2^_BW_ AD | 14.67 | 11.39 | 6.36 | 8.52 | 6.06 | 19.81 | 0.00 | . | 9.29 | 19.75 | 2.82 | 17.59 |
| σ^2^_BW_ Con | 37.30 | 10.44 | 24.26 | 8.32 | 43.17 | 14.15 | 36.17 | 12.86 | 38.89 | 13.66 | 30.71 | 11.89 |
| σ^2^ _BW_ DZ | 66.58 | 10.71 | 46.37 | 8.73 | 64.82 | 13.40 | 51.50 | 11.43 | 60.71 | 13.13 | 48.34 | 11.22 |
| σ^2^ _BW_ MZ | 101.11 | 11.93 | 74.28 | 9.19 | 98.48 | 13.94 | 74.51 | 10.90 | 99.16 | 13.81 | 75.55 | 10.93 |
| σ^2^_WI_ AD | 106.49 | 14.03 | 91.00 | 11.49 | 128.58 | 24.61 | 110.03 | 12.97 | 124.04 | 24.07 | 107.60 | 21.53 |
| σ^2^ _WI_ Con | 64.88 | 8.98 | 57.25 | 7.86 | 63.59 | 12.36 | 52.91 | 11.01 | 65.58 | 12.49 | 53.90 | 10.78 |
| σ^2^ _WI_ DZ | 49.50 | 5.89 | 49.73 | 5.94 | 51.08 | 8.55 | 47.47 | 7.87 | 52.45 | 8.75 | 48.60 | 8.03 |
| σ^2^ _WI_ MZ | 21.03 | 2.47 | 20.86 | 2.48 | 22.57 | 3.94 | 20.18 | 3.52 | 19.84 | 3.46 | 18.88 | 3.33 |
| Model Fit |  | |  | |  | |  | |  | |  | |
| -2 LL | 8644.1 | | 8442.2 | | 5477.5 | | 5345.4 | | 5461.2 | | 5332.2 | |
| AIC | 8678.1 | | 8480.2 | | 5511.5 | | 5381.4 | | 5495.2 | | 5370.2 | |
| N Individual/Sibships | 1172/712 | | 1172/712 | | 733/537 | | 733/537 | | 733/537 | | 733/537 | |

*Note*. VIQ=Verbal IQ; Unadj/Adj= Unadjusted/Adjusted for educational and occupational attainment; HPW=Log-transformed cognitive activity hours per week; Demand=Average cognitive hobby demand; Number= Number of cognitive hobbies; -2 LL= -2 log-likelihood; Random effects: σ2BW=between siblings and σ2WI=within siblings; subscript notes sibling type: AD=adoptive family siblings, Con=control family siblings, DZ=dizygotic twins, and MZ=monozygotic twins. Bolded parameters are significant p < .05

S8 Table. Model Parameters for SCI & IRR on VIQ, with and without socioeconomic adjustment

|  | SCI-  Unadj | | SCI-  Adj | | IRR-  Unadj | | IRR-  Adj | |
| --- | --- | --- | --- | --- | --- | --- | --- | --- |
|  | **B** | **se** | **B** | **se** | **B** | **se** | **B** | **se** |
| **Fixed Effects** |  |  |  |  |  |  |  |  |
| Intercept | **108.25** | 1.63 | **110.13** | 1.49 | **108.20** | 1.63 | **110.07** | 1.49 |
| Female | **-2.88** | 0.71 | **-3.92** | 0.64 | **-2.89** | 0.70 | **-3.91** | 0.64 |
| Age | 0.07 | 0.14 | -0.05 | 0.12 | 0.07 | 0.13 | -0.04 | 0.12 |
| Project | 1.90 | 1.46 | 1.68 | 1.30 | **1.94** | 1.44 | **1.70** | 1.30 |
| Adopted | **-4.87** | 1.08 | **-2.90** | 0.97 | **-5.06** | 1.07 | **-3.03** | 0.97 |
| Hispanic | -3.12 | 1.93 | -2.16 | 1.74 | -3.50 | 1.92 | -2.36 | 1.74 |
| White | 1.02 | 1.44 | 1.05 | 1.32 | 1.12 | 1.45 | 1.11 | 1.32 |
| Lives Together | -1.53 | 2.26 | -0.42 | 1.98 | -1.26 | 2.22 | -0.31 | 1.98 |
| Education | - | - | **1.43** | 0.11 | - | - | **1.41** | 0.11 |
| Occupation | - | - | 0.25 | 0.18 | - | - | 0.23 | 0.19 |
| SCI | 0.05 | 0.03 | 0.05 | 0.03 | - | - | - | - |
| IRR | - | - | - | - | **-8.42** | 2.49 | -2.83 | 2.38 |
| **Random Effects** |  |  |  |  |  |  |  |  |
| σ^2^_BW_ AD | 21.48 | 13.24 | 7.98 | 9.76 | 23.09 | 13.35 | 8.34 | 9.78 |
| σ^2^_BW_ Con | 42.16 | 11.35 | 29.12 | 9.14 | 36.61 | 11.08 | 27.57 | 9.11 |
| σ^2^ _BW_ DZ | 71.64 | 11.38 | 49.60 | 9.31 | 68.84 | 11.07 | 50.18 | 9.32 |
| σ^2^ _BW_ MZ | 103.94 | 12.18 | 76.33 | 9.36 | 99.60 | 11.89 | 75.01 | 9.27 |
| σ^2^_WI_ AD | 114.02 | 15.41 | 100.20 | 12.84 | 112.97 | 15.31 | 100.42 | 12.82 |
| σ^2^ _WI_ Con | 66.06 | 9.31 | 57.34 | 8.07 | 68.15 | 9.60 | 58.40 | 8.22 |
| σ^2^ _WI_ DZ | 51.69 | 6.15 | 53.12 | 6.33 | 51.08 | 6.06 | 52.62 | 6.27 |
| σ^2^ _WI_ MZ | 21.05 | 2.40 | 20.89 | 2.43 | 22.43 | 2.61 | 21.39 | 2.50 |
| Model Fit |  | |  | |  | |  | |
| -2 LL | 8732.7 | | 8533.2 | | 8725 | | 8535 | |
| AIC | 8766.7 | | 8571.2 | | 8759 | | 8573 | |
| N Individual/Sibships | 1176/712 | | 1176/712 | | 1176/712 | | 1176/712 | |

*Note.* VIQ=Verbal IQ; Unadj/Adj= Unadjusted/Adjusted for educational and occupational attainment; IRR=Index of Relative Rurality; SCI=Social Capital Index; -2 LL= -2 log-likelihood; Random effects: σ2BW=between siblings and σ2WI=within siblings; subscript notes sibling type: AD=adoptive family siblings, Con=control family siblings, DZ=dizygotic twins, and MZ=monozygotic twins. Bolded parameters are significant p < .05

S9 Table. Model Parameters for individual Engagement on PIQ, with and without socioeconomic adjustment

|  | HPW-  Unadj | | HPW-  Adj | | Demand- Unadj | | Demand-  Adj | | Number- Unadj | | Number-  Adj | |
| --- | --- | --- | --- | --- | --- | --- | --- | --- | --- | --- | --- | --- |
|  | **B** | **se** | **B** | **se** | **B** | **se** | **B** | **se** | **B** | **se** | **B** | **se** |
| **Fixed Effects** |  |  |  |  |  |  |  |  |  |  |  |  |
| Intercept | **107.53** | 2.03 | **109.25** | 1.96 | **109.94** | 2.49 | **111.62** | 2.42 | **112.04** | 2.45 | **113.66** | 2.38 |
| Female | 0.25 | 0.82 | -0.46 | 0.79 | 0.40 | 0.98 | -0.34 | 0.96 | -0.06 | 0.98 | -0.78 | 0.96 |
| Age | 0.02 | 0.17 | -0.08 | 0.16 | -0.06 | 0.21 | -0.15 | 0.21 | -0.07 | 0.22 | -0.16 | 0.21 |
| Project | **6.73** | 1.79 | **6.53** | 1.72 | **8.05** | 2.18 | **7.95** | 2.14 | **8.14** | 2.20 | **8.05** | 2.15 |
| Adopted | **-3.16** | 1.31 | -1.74 | 1.26 | **-3.53** | 1.69 | -2.44 | 1.64 | **-3.51** | 1.70 | -2.40 | 1.65 |
| Hispanic | -3.14 | 2.12 | -2.33 | 2.00 | -0.92 | 2.38 | -0.80 | 2.29 | -1.17 | 2.40 | -1.02 | 2.31 |
| White | -1.46 | 1.71 | -1.66 | 1.63 | -0.39 | 2.22 | -1.09 | 2.15 | -0.81 | 2.24 | -1.49 | 2.16 |
| Live Together | -4.49 | 2.33 | -3.47 | 2.17 | -3.79 | 2.82 | -2.99 | 2.69 | -4.19 | 2.83 | -3.36 | 2.69 |
| Education | - | - | **1.04** | 0.14 | - | - | **0.93** | 0.18 | - | - | **0.91** | 0.18 |
| Occupation | - | - | 0.42 | 0.25 | - | - | 0.31 | 0.33 | - | - | 0.36 | 0.33 |
| HPW | **2.88** | 0.49 | **2.76** | 0.47 | - | - | - | - | - | - | - | - |
| Demand | - | - | - | - | **7.04** | 1.97 | **6.96** | 1.93 | - | - | - | - |
| Number | - | - | - | - | - | - | - | - | 0.78 | 1.12 | 0.64 | 1.10 |
| **Random Effects** |  |  |  |  |  |  |  |  |  |  |  |  |
| σ^2^_BW_ AD | 15.99 | 16.99 | 3.38 | 14.97 | 4.82 | 27.30 | 3.76 | 28.51 | 13.95 | 28.79 | 13.94 | 29.98 |
| σ^2^_BW_ Con | 73.27 | 21.59 | 62.73 | 20.26 | 81.64 | 28.34 | 77.86 | 28.31 | 76.46 | 28.34 | 73.92 | 28.20 |
| σ^2^ _BW_ DZ | 46.14 | 12.25 | 34.30 | 11.50 | 23.53 | 14.60 | 20.50 | 13.99 | 24.78 | 15.00 | 21.27 | 14.35 |
| σ^2^ _BW_ MZ | 103.86 | 14.03 | 83.70 | 12.20 | 90.26 | 17.76 | 72.52 | 16.18 | 85.35 | 17.85 | 67.48 | 16.39 |
| σ^2^_WI_ AD | 170.77 | 22.08 | 164.50 | 20.94 | 194.31 | 35.46 | 177.72 | 35.27 | 189.19 | 35.67 | 171.30 | 35.31 |
| σ^2^ _WI_ Con | 119.09 | 17.60 | 120.32 | 17.52 | 102.02 | 22.78 | 103.79 | 23.19 | 106.29 | 23.60 | 106.82 | 23.67 |
| σ^2^ _WI_ DZ | 95.12 | 11.17 | 99.38 | 11.69 | 103.21 | 16.05 | 101.95 | 15.74 | 104.91 | 16.34 | 103.69 | 16.04 |
| σ^2^ _WI_ MZ | 47.22 | 5.45 | 47.22 | 5.49 | 60.74 | 10.57 | 62.32 | 10.88 | 65.87 | 11.43 | 68.36 | 11.88 |
| Model Fit |  | |  | |  | |  | |  | |  | |
| -2 LL | 9174.1 | | 9096.2 | | 5754.2 | | 5716.4 | | 5766.1 | | 5728.6 | |
| AIC | 9208.1 | | 9134.2 | | 5788.2 | | 5754.4 | | 5800.1 | | 5766.6 | |
| *N*  Individual/Sibships | 1172/712 | | 1172/712 | | 733/537 | | 733/537 | | 733/537 | | 733/537 | |

*Note*. PIQ=Performance IQ; Unadj/Adj= Unadjusted/Adjusted for educational and occupational attainment; HPW=Log-transformed cognitive activity hours per week; Demand=Average cognitive hobby demand; Number= Number of cognitive hobbies; -2 LL= -2 log-likelihood; Random effects: σ2BW=between siblings and σ2WI=within siblings; subscript notes sibling type: AD=adoptive family siblings, Con=control family siblings, DZ=dizygotic twins, and MZ=monozygotic twins. Bolded parameters are significant p < .05

S10 Table. Model Parameters for SCI & IRR on PIQ, with and without socioeconomic adjustment

|  | SCI-  Unadj | | SCI-  Adj | | IRR-  Unadj | | IRR-  Adj | |
| --- | --- | --- | --- | --- | --- | --- | --- | --- |
|  | **B** | **se** | **B** | **se** | **B** | **se** | **B** | **se** |
| **Fixed Effects** |  |  |  |  |  |  |  |  |
| Intercept | **111.68** | 1.95 | **113.22** | 1.87 | **111.63** | 1.94 | **113.16** | 1.87 |
| Female | -0.09 | 0.83 | -0.79 | 0.80 | -0.09 | 0.83 | -0.78 | 0.80 |
| Age | 0.00 | 0.17 | -0.10 | 0.16 | 0.01 | 0.17 | -0.10 | 0.16 |
| Project | **6.99** | 1.81 | **6.77** | 1.73 | **6.94** | 1.80 | **6.76** | 1.73 |
| Adopted | **-3.39** | 1.34 | -1.96 | 1.29 | **-3.52** | 1.34 | -2.04 | 1.29 |
| Hispanic | -3.18 | 2.16 | -2.31 | 2.04 | -3.40 | 2.14 | -2.43 | 2.04 |
| White | -1.14 | 1.75 | -1.33 | 1.66 | -1.03 | 1.74 | -1.27 | 1.66 |
| Lives Together | -3.94 | 2.37 | -2.93 | 2.21 | -3.71 | 2.35 | -2.85 | 2.21 |
| Education | - | - | **1.04** | 0.14 | - | - | **1.03** | 0.14 |
| Occupation | - | - | 0.47 | 0.25 | - | - | 0.46 | 0.25 |
| SCI | 0.03 | 0.04 | 0.03 | 0.04 | - | - | - | - |
| IRR | - | - | - | - | **-7.44** | 3.20 | -2.06 | 3.19 |
| **Random Effects** | |  |  |  |  |  |  |  |
| σ^2^_BW_ AD | 21.95 | 18.29 | 5.90 | 15.80 | 23.14 | 18.39 | 6.66 | 15.87 |
| σ^2^_BW_ Con | 73.94 | 22.51 | 63.25 | 21.07 | 69.71 | 22.14 | 61.83 | 20.88 |
| σ^2^ _BW_ DZ | 48.89 | 12.66 | 36.03 | 11.88 | 47.10 | 12.44 | 36.31 | 11.86 |
| σ^2^ _BW_ MZ | 108.07 | 14.38 | 87.43 | 12.51 | 105.38 | 14.24 | 87.03 | 12.51 |
| σ^2^_WI_ AD | 176.91 | 23.07 | 171.06 | 21.78 | 175.41 | 22.98 | 170.69 | 21.77 |
| σ^2^ _WI_ Con | 119.13 | 18.01 | 119.76 | 17.86 | 121.71 | 18.27 | 120.84 | 17.93 |
| σ^2^ _WI_ DZ | 98.50 | 11.51 | 103.70 | 12.13 | 98.12 | 11.43 | 103.28 | 12.08 |
| σ^2^ _WI_ MZ | 46.65 | 5.31 | 46.99 | 5.40 | 47.69 | 5.47 | 47.19 | 5.44 |
| Model Fit |  | |  | |  | |  | |
| -2 LL | 9235.9 | | 9157.2 | | 9231.3 | | 9157.5 | |
| AIC | 9269.9 | | 9195.2 | | 9265.3 | | 9195.5 | |
| *N*  Individual/Sibships | 1176/712 | | 1176/712 | | 1176/712 | | 1176/712 | |

*Note.* PIQ=Performance IQ; Unadj/Adj= Unadjusted/Adjusted for educational and occupational attainment; IRR=Index of Relative Rurality; SCI=Social Capital Index; -2 LL= -2 log-likelihood; Random effects: σ2BW=between siblings and σ2WI=within siblings; subscript notes sibling type: AD=adoptive family siblings, Con=control family siblings, DZ=dizygotic twins, and MZ=monozygotic twins. Bolded parameters are significant p < .05

| S11 Table. Model Parameters for HPW cognitive on FSIQ | | | | | | | | |
| --- | --- | --- | --- | --- | --- | --- | --- | --- |
|  | Model 1 | | Model 2 | | Model 3 | | Model 4 | |
|  | **B** | **se** | **B** | **se** | **B** | **se** | **B** | **se** |
| **Fixed Effects** |  |  |  |  |  |  |  |  |
| Intercept | **107.78** | 1.59 | **107.73** | 1.59 | **107.73** | 1.59 | 107.55 | 1.59 |
| Female | **-2.58** | 0.66 | **-2.56** | 0.65 | **-2.56** | 0.66 | **-2.56** | 0.65 |
| Age | -0.05 | 0.13 | -0.05 | 0.13 | -0.05 | 0.13 | -0.06 | 0.13 |
| Project | **3.88** | 1.38 | **3.86** | 1.38 | **3.86** | 1.38 | **3.99** | 1.37 |
| Adopted | **-2.64** | 1.01 | **-2.72** | 1.01 | **-2.72** | 1.01 | **-2.79** | 1.01 |
| Hispanic | -2.24 | 1.70 | -2.34 | 1.70 | -2.33 | 1.70 | -2.22 | 1.69 |
| White | -0.06 | 1.33 | -0.06 | 1.33 | -0.06 | 1.33 | -0.01 | 1.32 |
| Live Together | -2.10 | 1.90 | -2.04 | 1.89 | -2.04 | 1.89 | -2.13 | 1.89 |
| Education | **1.31** | 0.11 | **1.29** | 0.11 | **1.29** | 0.11 | **1.28** | 0.11 |
| Occupation | 0.37 | 0.19 | 0.36 | 0.19 | 0.36 | 0.19 | 0.38 | 0.19 |
| HPW Cognitive | **3.03** | 0.37 | **3.07** | 0.37 | **3.07** | 0.37 | **3.15** | 0.37 |
| Social Capital | 0.03 | 0.03 | 0.03 | 0.03 | 0.03 | 0.03 | 0.04 | 0.03 |
| IRR | - | - | -3.72 | 2.47 | -2.76 | 5.84 | -2.11 | 2.54 |
| HPW Cognitive * IRR | - | - | - | - | -0.61 | 3.39 | - | - |
| SCI* IRR | - | - | - | - | - | - | **-0.48** | 0.18 |
| **Random Effects** |  |  |  |  |  |  |  |  |
| σ^2^_BW_ AD | 2.40 | 9.32 | 3.11 | 9.43 | 3.02 | 9.44 | 3.04 | 9.38 |
| σ^2^_BW_ Control | 38.88 | 12.27 | 37.50 | 12.23 | 37.39 | 12.23 | 35.94 | 12.07 |
| σ^2^ _BW_ DZ | 38.06 | 8.72 | 37.63 | 8.62 | 37.61 | 8.63 | 37.02 | 8.53 |
| σ^2^ _BW_ MZ | 69.68 | 8.91 | 68.73 | 8.87 | 68.75 | 8.87 | 69.21 | 8.9 |
| σ^2^_WI_ AD | 109.54 | 13.48 | 109.16 | 13.49 | 109.22 | 13.49 | 108.1 | 13.38 |
| σ^2^ _WI_ Control | 69.37 | 10.29 | 70.31 | 10.43 | 70.34 | 10.43 | 71.37 | 10.53 |
| σ^2^ _WI_ DZ | 59.09 | 7.04 | 58.44 | 6.97 | 58.48 | 6.98 | 57.88 | 6.91 |
| σ^2^ _WI_ MZ | 22.29 | 2.65 | 22.65 | 2.71 | 22.66 | 2.71 | 22.46 | 2.68 |
| Model Fit |  | | M1-M2 | | M2-M3 | | M2-M4 | |
| -2 LL | 8568.5 | | 8566.3 | | 8566.3 | | 8559.5 | |
| AIC | 8608.5 | | 8608.3 | | 8610.3 | | 8603.5 | |
| Δχ^2^ |  | | 2.2 | | 0 | | 6.8 | |
| df |  | | 1 | | 1 | | 1 | |
| *p* |  | | 0.14 | | -- | | 0.01 | |
| *Note*. IRR=Index of Relative Rurality; SCI=Social Capital Index; and IQ scores; FSIQ=Full-scale IQ; -2 LL= -2 log-likelihood; Random effects: σ^2^BW=between siblings and σ^2^WI=within siblings; subscript notes sibling type: AD=adoptive family siblings, Con=control family siblings, DZ=dizygotic twins, and MZ=monozygotic twins. Bolded parameters are significant p < .05, Individual N=1172; Sibships N=712 | | | | | | | | |

| S12 Table. Model Parameters for cognitive demands of hobbies on FSIQ | | | | | | | | |
| --- | --- | --- | --- | --- | --- | --- | --- | --- |
|  | Model 1 | | Model 2 | | Model 3 | | Model 4 | |
|  | **B** | **se** | **B** | **se** | **B** | **se** | **B** | **se** |
| **Fixed Effects** |  |  |  |  |  |  |  |  |
| Intercept | **110.43** | 2.03 | **110.32** | 2.03 | **110.27** | 2.03 | **110.05** | 2.02 |
| Female | **-2.90** | 0.81 | **-2.87** | 0.81 | **-2.90** | 0.81 | **-2.87** | 0.80 |
| Age | -0.14 | 0.17 | -0.13 | 0.17 | -0.13 | 0.17 | -0.15 | 0.17 |
| Project | **5.34** | 1.74 | **5.33** | 1.74 | **5.36** | 1.74 | **5.49** | 1.74 |
| Adopted | **-2.83** | 1.33 | **-2.96** | 1.33 | **-2.94** | 1.34 | **-3.00** | 1.33 |
| Hispanic | -0.43 | 1.97 | -0.51 | 1.95 | -0.51 | 1.95 | -0.23 | 1.94 |
| White | 0.47 | 1.81 | 0.62 | 1.81 | 0.73 | 1.81 | 0.97 | 1.80 |
| Live Together | -1.62 | 2.33 | -1.59 | 2.31 | -1.62 | 2.31 | -1.70 | 2.30 |
| Education | **1.33** | 0.15 | **1.29** | 0.15 | **1.29** | 0.15 | **1.27** | 0.15 |
| Occupation | 0.18 | 0.26 | 0.16 | 0.26 | 0.13 | 0.26 | 0.20 | 0.26 |
| Demand | **7.51** | 1.54 | **7.37** | 1.54 | **7.28** | 1.54 | **7.13** | 1.53 |
| Number | **2.96** | 0.85 | **2.98** | 0.85 | **2.99** | 0.85 | **3.08** | 0.85 |
| Social Capital | 0.06 | 0.04 | 0.07 | 0.04 | **0.08** | 0.04 | **0.10** | 0.04 |
| IRR | - | - | -5.22 | 3.15 | **-9.34** | 4.42 | -3.8 | 3.17 |
| Demand* IRR | - | - | - | - | 16.59 | 12.37 | - | - |
| SCI* IRR | - | - | - | - | - | - | **-0.69** | 0.26 |
| **Random Effects** |  |  |  |  |  |  |  |  |
| σ^2^_BW_ AD | 0.00 | . | 0.00 | . | 0.00 | . | 0.00 | . |
| σ^2^_BW_ Con | 45.53 | 19.21 | 44.06 | 19.31 | 45.92 | 19.41 | 48.15 | 19.11 |
| σ^2^ _BW_ DZ | 30.43 | 10.44 | 29.07 | 10.24 | 28.48 | 10.10 | 26.22 | 9.97 |
| σ^2^ _BW_ MZ | 65.24 | 10.52 | 63.87 | 10.45 | 65.10 | 10.55 | 64.74 | 10.49 |
| σ^2^_WI_ AD | 121.69 | 14.39 | 122.24 | 14.47 | 123.66 | 14.68 | 121.25 | 14.34 |
| σ^2^ _WI_ Con | 68.61 | 16.22 | 69.41 | 16.46 | 68.31 | 16.27 | 66.79 | 15.74 |
| σ^2^ _WI_ DZ | 59.61 | 9.50 | 59.54 | 9.44 | 59.04 | 9.34 | 59.34 | 9.41 |
| σ^2^ _WI_ MZ | 26.32 | 4.55 | 26.99 | 4.68 | 26.01 | 4.57 | 26.53 | 4.59 |
| Model Fit |  | |  | |  | |  | |
| -2 LL | 5406.1 | | 5403.4 | | 5401.7 | | 5396.5 | |
| AIC | 5446.1 | | 5445.4 | | 5445.7 | | 5440.5 | |
| Δχ^2^ |  | | 2.7 | | 1.7 | | 6.9 | |
| df |  | | 1 | | 1 | | 1 | |
| *p* |  | | 0.10 | | 0.19 | | 0.01 | |
| *Note*. IRR=Index of Relative Rurality; SCI=Social Capital Index; and IQ scores; FSIQ=Full-scale IQ; Demand=Average cognitive demand of reported hobbies; Number= Number of cognitive hobbies; -2 LL= -2 log-likelihood; Random effects: σ^2^BW=between siblings and σ^2^WI=within siblings; subscript notes sibling type: AD=adoptive family siblings, Con=control family siblings, DZ=dizygotic twins, and MZ=monozygotic twins. Bolded parameters are significant p < .05, Individual N=733; Sibships N=537 | | | | | | | | |

| S13 Table. Model Parameters for HPW cognitive on VIQ | | | | | | | | |
| --- | --- | --- | --- | --- | --- | --- | --- | --- |
|  | Model 1 | | Model 2 | | Model 3 | | Model 4 | |
|  | **B** | **se** | **B** | **se** | **B** | **se** | **B** | **se** |
| **Fixed Effects** |  |  |  |  |  |  |  |  |
| Intercept | **106.03** | 1.52 | **105.95** | 1.52 | **105.95** | 1.52 | **105.88** | 1.52 |
| Female | **-3.54** | 0.62 | **-3.53** | 0.62 | **-3.51** | 0.62 | **-3.52** | 0.62 |
| Age | -0.02 | 0.12 | -0.02 | 0.12 | -0.02 | 0.12 | -0.02 | 0.12 |
| Project | 1.31 | 1.26 | 1.30 | 1.26 | 1.32 | 1.26 | 1.35 | 1.26 |
| Adopted | **-2.69** | 0.92 | **-2.79** | 0.92 | **-2.82** | 0.92 | **-2.82** | 0.92 |
| Hispanic | -2.17 | 1.69 | -2.29 | 1.69 | -2.28 | 1.69 | -2.24 | 1.69 |
| White | 0.70 | 1.27 | 0.71 | 1.27 | 0.69 | 1.27 | 0.72 | 1.27 |
| Live Together | -1.05 | 1.94 | -0.97 | 1.93 | -0.99 | 1.93 | -1.01 | 1.93 |
| Education | **1.39** | 0.11 | **1.36** | 0.11 | **1.36** | 0.11 | **1.36** | 0.11 |
| Occupation | 0.27 | 0.18 | 0.26 | 0.18 | 0.27 | 0.18 | 0.27 | 0.18 |
| HPW Cognitive | **2.87** | 0.35 | **2.91** | 0.35 | **2.91** | 0.35 | **2.95** | 0.35 |
| Social Capital | 0.03 | 0.03 | 0.03 | 0.03 | 0.04 | 0.03 | 0.04 | 0.03 |
| IRR | - | - | -4.52 | 2.34 | 0.86 | 5.52 | -3.75 | 2.42 |
| HPW Cognitive * IRR | - | - | - | - | -3.44 | 3.20 | - | - |
| SCI* IRR | - | - | - | - | - | - | -0.22 | 0.17 |
| **Random Effects** |  |  |  |  |  |  |  |  |
| σ^2^_BW_ AD | 6.66 | 8.60 | 7.72 | 8.75 | 7.08 | 8.72 | 7.76 | 8.73 |
| σ^2^_BW_ Con | 24.77 | 8.35 | 23.11 | 8.29 | 22.44 | 8.26 | 23.00 | 8.25 |
| σ^2^ _BW_ DZ | 45.67 | 8.70 | 45.10 | 8.58 | 45.03 | 8.58 | 44.88 | 8.57 |
| σ^2^ _BW_ MZ | 74.53 | 9.21 | 73.50 | 9.14 | 73.81 | 9.18 | 73.47 | 9.14 |
| σ^2^_WI_ AD | 90.75 | 11.51 | 90.30 | 11.51 | 90.72 | 11.54 | 89.76 | 11.46 |
| σ^2^ _WI_ Con | 56.72 | 7.81 | 57.72 | 7.94 | 58.01 | 7.98 | 57.85 | 7.94 |
| σ^2^ _WI_ DZ | 49.95 | 5.97 | 49.19 | 5.88 | 49.25 | 5.90 | 49.19 | 5.89 |
| σ^2^ _WI_ MZ | 20.79 | 2.47 | 21.11 | 2.52 | 21.04 | 2.51 | 21.13 | 2.52 |
| Model Fit | M1 | | M1-M2 | | M2-M3 | | M2-M4 | |
| -2 LL | 8441.0 | | 8437.3 | | 8436.2 | | 8435.7 | |
| AIC | 8481.0 | | 8479.3 | | 8480.2 | | 8479.7 | |
| Δχ^2^ |  | | 3.7 | | 1.1 | | 1.6 | |
| df |  | | 1 | | 1 | | 1 | |
| *p* |  | | 0.05 | | 0.29 | | 0.21 | |
| *Note*. IRR=Index of Relative Rurality; SCI=Social Capital Index; and IQ scores; PIQ=Performance IQ; -2 LL= -2 log-likelihood; Random effects: σ^2^BW=between siblings and σ^2^WI=within siblings; subscript notes sibling type: AD=adoptive family siblings, Con=control family siblings, DZ=dizygotic twins, and MZ=monozygotic twins. Bolded parameters are significant p < .05, Individual N=1172; Sibships N=712 | | | | | | | | |

| S14 Table. Model Parameters for cognitive demands of hobbies on VIQ | | | | | | | | |
| --- | --- | --- | --- | --- | --- | --- | --- | --- |
|  | Model 1 | | Model 2 | | Model 3 | | Model 4 | |
|  | **B** | **se** | **B** | **se** | **B** | **se** | **B** | **se** |
| **Fixed Effects** |  |  |  |  |  |  |  |  |
| Intercept | **108.78** | 1.97 | **108.64** | 1.97 | **108.63** | 1.97 | **108.56** | 1.96 |
| Female | **-4.15** | 0.78 | **-4.13** | 0.78 | **-4.14** | 0.78 | **-4.14** | 0.78 |
| Age | -0.13 | 0.16 | -0.12 | 0.16 | -0.12 | 0.16 | -0.13 | 0.16 |
| Project | *2.66* | 1.60 | 2.65 | 1.60 | 2.66 | 1.60 | 2.70 | 1.60 |
| Adopted | **-2.48** | 1.22 | **-2.62** | 1.22 | **-2.62** | 1.22 | **-2.64** | 1.22 |
| Hispanic | -0.56 | 1.96 | -0.63 | 1.95 | -0.62 | 1.95 | -0.50 | 1.95 |
| White | 1.26 | 1.76 | 1.43 | 1.76 | 1.47 | 1.77 | 1.58 | 1.76 |
| Live Together | -0.48 | 2.38 | -0.45 | 2.37 | -0.45 | 2.37 | -0.49 | 2.36 |
| Education | **1.48** | 0.14 | **1.43** | 0.14 | **1.43** | 0.14 | **1.42** | 0.14 |
| Occupation | 0.06 | 0.24 | 0.04 | 0.24 | 0.03 | 0.24 | 0.06 | 0.24 |
| Demand | **5.47** | 1.41 | **5.37** | 1.41 | **5.33** | 1.41 | **5.22** | 1.42 |
| Number | **4.06** | 0.77 | **4.06** | 0.77 | **4.06** | 0.77 | **4.12** | 0.77 |
| Social Capital | 0.06 | 0.04 | 0.07 | 0.04 | 0.07 | 0.04 | **0.08** | 0.04 |
| IRR | - | - | -5.52 | 2.88 | -6.63 | 4.07 | -4.86 | 2.91 |
| Demand* IRR | - | - | - | - | 4.37 | 11.28 | - | - |
| SCI* IRR | - | - | - | - | - | - | -0.35 | 0.24 |
| **Random Effects** |  |  |  |  |  |  |  |  |
| σ^2^_BW_ AD | 0.00 | . | 0.00 | . | 0.00 | . | 0.00 | . |
| σ^2^_BW_ Con | 30.64 | 12.49 | 29.03 | 12.47 | 29.26 | 12.49 | 29.86 | 12.52 |
| σ^2^ _BW_ DZ | 46.00 | 10.68 | 44.2 | 10.51 | 44.02 | 10.49 | 43.06 | 10.43 |
| σ^2^ _BW_ MZ | 73.30 | 10.60 | 72.59 | 10.53 | 72.79 | 10.56 | 72.49 | 10.52 |
| σ^2^_WI_ AD | 107.91 | 12.71 | 108.84 | 12.84 | 109.2 | 12.92 | 108.39 | 12.79 |
| σ^2^ _WI_ Con | 55.99 | 11.44 | 56.7 | 11.59 | 56.55 | 11.57 | 56.2 | 11.51 |
| σ^2^ _WI_ DZ | 45.44 | 7.55 | 45.5 | 7.54 | 45.48 | 7.54 | 45.59 | 7.57 |
| σ^2^ _WI_ MZ | 18.42 | 3.24 | 18.59 | 3.27 | 18.45 | 3.26 | 18.62 | 3.27 |
| Model Fit | M1 | | M1-M2 | | M2-M3 | | M2-M4 | |
| -2 LL | 5314.9 | | 5311.3 | | 5311.2 | | 5309.2 | |
| AIC | 5354.9 | | 5353.3 | | 5355.2 | | 5353.2 | |
| Δχ^2^ |  | | 3.6 | | 0.1 | | 2.1 | |
| df |  | | 1 | | 1 | | 1 | |
| *p* |  | | 0.06 | | 0.75 | | 0.15 | |
| *Note*. IRR=Index of Relative Rurality; SCI=Social Capital Index; and IQ scores; PIQ=Verbal IQ; Demand=Average cognitive demand of reported hobbies; Number= Number of cognitive hobbies; -2 LL= -2 log-likelihood; Random effects: σ^2^BW=between siblings and σ^2^WI=within siblings; subscript notes sibling type: AD=adoptive family siblings, Con=control family siblings, DZ=dizygotic twins, and MZ=monozygotic twins. Bolded parameters are significant p < .05, Individual N=733; Sibships N=537 | | | | | | | | |

| S15 Table. Model Parameters for HPW cognitive on PIQ | | | | | | | | |
| --- | --- | --- | --- | --- | --- | --- | --- | --- |
|  | Model 1 | | Model 2 | | Model 3 | | Model 4 | |
|  | **B** | **se** | **B** | **se** | **B** | **se** | **B** | **se** |
| **Fixed Effects** |  |  |  |  |  |  |  |  |
| Intercept | **109.29** | 1.96 | **109.23** | 1.96 | **109.23** | 1.96 | **108.99** | 1.95 |
| Female | -0.47 | 0.79 | -0.46 | 0.79 | -0.47 | 0.79 | -0.47 | 0.79 |
| Age | -0.09 | 0.16 | -0.08 | 0.16 | -0.08 | 0.16 | -0.09 | 0.16 |
| Project | **6.53** | 1.72 | **6.51** | 1.72 | **6.49** | 1.72 | **6.66** | 1.71 |
| Adopted | -1.72 | 1.26 | **-1.77** | 1.27 | **-1.74** | 1.27 | **-1.87** | 1.26 |
| Hispanic | -2.27 | 2.00 | -2.32 | 2.00 | -2.35 | 2.00 | -2.15 | 2.00 |
| White | -1.68 | 1.63 | -1.65 | 1.63 | -1.65 | 1.63 | -1.54 | 1.63 |
| Live Together | -3.49 | 2.17 | -3.43 | 2.16 | -3.43 | 2.16 | -3.56 | 2.16 |
| Education | **1.04** | 0.14 | **1.02** | 0.14 | **1.02** | 0.14 | 1.01 | 0.14 |
| Occupation | 0.42 | 0.25 | **0.41** | 0.25 | **0.40** | 0.25 | 0.44 | 0.25 |
| HPW Cognitive | **2.74** | 0.48 | **2.76** | 0.48 | **2.76** | 0.48 | **2.86** | 0.48 |
| Social Capital | 0.02 | 0.04 | 0.02 | 0.04 | 0.02 | 0.04 | 0.04 | 0.04 |
| IRR | - | - | -2.78 | 3.17 | -8.02 | 7.61 | -0.54 | 3.26 |
| HPW Cognitive * IRR | - | - | - | - | 3.35 | 4.42 | - | - |
| SCI* IRR | - | - | - | - | - | - | **-0.65** | 0.24 |
| **Random Effects** |  |  |  |  |  |  |  |  |
| σ^2^_BW_ AD | 3.21 | 14.97 | 3.61 | 15.02 | 4.00 | 15.05 | 3.15 | 14.93 |
| σ^2^_BW_ Con | 63.70 | 20.41 | 63.23 | 20.35 | 63.34 | 20.41 | 59.21 | 20.11 |
| σ^2^ _BW_ DZ | 33.97 | 11.50 | 33.93 | 11.44 | 33.84 | 11.41 | 33.27 | 11.26 |
| σ^2^ _BW_ MZ | 83.66 | 12.19 | 83.07 | 12.18 | 83.13 | 12.18 | 84.22 | 12.27 |
| σ^2^_WI_ AD | 164.58 | 20.95 | 164.23 | 20.94 | 163.89 | 20.92 | 163.14 | 20.82 |
| σ^2^ _WI_ Con | 119.56 | 17.49 | 120.07 | 17.53 | 120.33 | 17.58 | 122.76 | 17.86 |
| σ^2^ _WI_ DZ | 99.57 | 11.72 | 99.06 | 11.66 | 98.68 | 11.63 | 97.59 | 11.49 |
| σ^2^ _WI_ MZ | 47.24 | 5.50 | 47.55 | 5.55 | 47.51 | 5.54 | 47.17 | 5.49 |
| Model Fit |  | | M1-M2 | | M2-M3 | | M2-M4 | |
| -2 LL | 9095.8 | | 9095.1 | | 9094.5 | | 9087.7 | |
| AIC | 9135.8 | | 9137.1 | | 9138.5 | | 9131.7 | |
| Δχ^2^ |  | | 0.7 | | 0.6 | | 7.4 | |
| df |  | | 1 | | 1 | | 1 | |
| *p* |  | | 0.40 | | 0.44 | | 0.01 | |
| *Note*. IRR=Index of Relative Rurality; SCI=Social Capital Index; and IQ scores; PIQ=Performance IQ; -2 LL= -2 log-likelihood; Random effects: σ^2^BW=between siblings and σ^2^WI=within siblings; subscript notes sibling type: AD=adoptive family siblings, Con=control family siblings, DZ=dizygotic twins, and MZ=monozygotic twins. Bolded parameters are significant p < .05, Individual N=1172; Sibships N=712 | | | | | | | | |

| S16 Table. Model Parameters for cognitive demands of hobbies on PIQ | | | | | | | | |
| --- | --- | --- | --- | --- | --- | --- | --- | --- |
|  | Model 1 | | Model 2 | | Model 3 | | Model 4 | |
|  | **B** | **se** | **B** | **se** | **B** | **se** | **B** | **se** |
| **Fixed Effects** |  |  |  |  |  |  |  |  |
| Intercept | **111.72** | 2.41 | **111.60** | 2.41 | **111.57** | 2.41 | **111.16** | 2.39 |
| Female | -0.45 | 0.95 | -0.44 | 0.95 | -0.49 | 0.95 | -0.44 | 0.94 |
| Age | -0.12 | 0.21 | -0.11 | 0.21 | -0.11 | 0.21 | -0.13 | 0.21 |
| Project | **7.67** | 2.14 | **7.69** | 2.14 | **7.72** | 2.15 | **7.86** | 2.14 |
| Adopted | -2.40 | 1.64 | -2.50 | 1.64 | -2.47 | 1.65 | -2.54 | 1.64 |
| Hispanic | -0.54 | 2.28 | -0.58 | 2.28 | -0.63 | 2.27 | -0.16 | 2.25 |
| White | -1.12 | 2.13 | -0.98 | 2.14 | -0.84 | 2.14 | -0.41 | 2.12 |
| Live Together | -3.08 | 2.67 | -3.06 | 2.66 | -3.12 | 2.65 | -3.24 | 2.65 |
| Education | **0.91** | 0.18 | **0.88** | 0.18 | **0.88** | 0.18 | **0.85** | 0.18 |
| Occupation | 0.32 | 0.33 | 0.30 | 0.33 | 0.27 | 0.32 | 0.36 | 0.32 |
| Demand | **7.04** | 1.94 | **6.86** | 1.94 | **6.82** | 1.93 | **6.62** | 1.93 |
| Number | 0.91 | 1.09 | 0.91 | 1.09 | 0.95 | 1.09 | 1.05 | 1.08 |
| Social Capital | 0.06 | 0.05 | 0.07 | 0.05 | 0.08 | 0.05 | **0.11** | 0.05 |
| IRR | - | - | -3.98 | 3.97 | **-11.18** | 5.58 | -1.74 | 4.00 |
| Demand* IRR | - | - | - | - | 29.15 | 15.74 | - | - |
| SCI* IRR | - | - | - | - | - | - | **-0.95** | 0.32 |
| **Random Effects** |  |  |  |  |  |  |  |  |
| σ^2^_BW_ AD | 4.91 | 28.33 | 5.03 | 28.15 | 4.80 | 28.33 | 6.67 | 27.79 |
| σ^2^_BW_ Con | 76.80 | 28.50 | 76.48 | 28.46 | 80.30 | 28.98 | 82.61 | 28.42 |
| σ^2^ _BW_ DZ | 17.39 | 13.89 | 17.16 | 13.75 | 16.30 | 13.40 | 12.54 | 13.26 |
| σ^2^ _BW_ MZ | 71.13 | 16.11 | 69.56 | 16.11 | 72.30 | 16.24 | 73.20 | 16.30 |
| σ^2^_WI_ AD | 177.04 | 34.98 | 176.81 | 34.81 | 179.22 | 35.18 | 173.39 | 34.12 |
| σ^2^ _WI_ Con | 104.56 | 23.58 | 104.86 | 23.60 | 102.82 | 23.47 | 101.06 | 22.74 |
| σ^2^ _WI_ DZ | 103.08 | 15.88 | 102.84 | 15.77 | 101.09 | 15.45 | 102.17 | 15.62 |
| σ^2^ _WI_ MZ | 63.19 | 11.03 | 64.10 | 11.22 | 61.68 | 10.89 | 62.52 | 10.92 |
| Model Fit |  | | M1-M2 | | M2-M3 | | M2-M4 | |
| -2 LL | 5713.7 | | 5712.8 | | 5709.5 | | 5704.5 | |
| AIC | 5755.7 | | 5756.8 | | 5755.5 | | 5750.5 | |
| Δχ^2^ |  | | 0.9 | | 3.3 | | 8.3 | |
| df |  | | 1 | | 1 | | 1 | |
| *p* |  | | 0.34 | | 0.07 | | 0.004 | |
| *Note*. IRR=Index of Relative Rurality; SCI=Social Capital Index; and IQ scores; PIQ=Performance IQ; Demand=Average cognitive demand of reported hobbies; Number= Number of cognitive hobbies; -2 LL= -2 log-likelihood; Random effects: σ^2^BW=between siblings and σ^2^WI=within siblings; subscript notes sibling type: AD=adoptive family siblings, Con=control family siblings, DZ=dizygotic twins, and MZ=monozygotic twins. Bolded parameters are significant p < .05, Individual N=733; Sibships N=537 | | | | | | | | |

S17 Table. Sensitivity Analysis: Parameter Estimates and Model Fit for VIQ.

|  | **Base** | | Add Year 16 IQ | |
| --- | --- | --- | --- | --- |
|  | **B** | **se** | **B** | **se** |
| **HPW Cognitive** |  |  |  |  |
| Intercept | **105.99** | 1.56 | **103.70** | 1.01 |
| HPW | **3.15** | 0.37 | **1.66** | 0.26 |
| SCI | 0.03 | 0.03 | -0.003 | 0.02 |
| IRR | **-5.19** | 2.42 | 0.58 | 1.69 |
| Year diff |  |  | **0.57** | 0.15 |
| IQ16 |  |  | **0.67** | 0.02 |
| Model Fit |  |  |  |  |
| -2 LL | 7778.9 | | 7223.0 | |
| AIC | 7820.9 | | 7267.0 | |
| Model Comparison |  | |  |  |
| Δχ^2^ (df) | -- | | 555.9 (2) | |
| *P* | -- | | <.0001 | |
| **Cognitive Demand** |  |  |  |  |
| Intercept | **108.63** | 2.02 | **105.82** | 1.24 |
| Demand | **5.84** | 1.50 | 1.15 | 1.02 |
| Number | **4.30** | 0.82 | **2.08** | 0.57 |
| SCI | 0.06 | 0.04 | -0.001 | 0.02 |
| IRR | **-6.76** | 3.01 | -0.67 | 2.08 |
| Year diff |  |  | **0.48** | 0.17 |
| IQ16 |  |  | **0.68** | 0.02 |
| Model Fit |  |  |  |  |
| -2 LL | 4918.6 | | 4510.0 | |
| AIC | 4960.6 | | 4556.0 | |
| Model Comparison |  | |  | |
| Δχ^2^ (df) | -- | | 408.6 (2) | |
| *P* | -- | | <.0001 | |

*Note*. VIQ=Verbal IQ; HPW=Log-transformed cognitive hours per week; SCI=Social Capital Index; IRR=Index of Relative Rurality; Demand=Average cognitive demand of reported hobbies; Number= Number of cognitive hobbies; Year diff= Year difference between CATSLife and Year 16 assessments, centered at 15 years; IQ16= Adolescent VIQ assessed at approximately 16 years of age, centered at 100; -2 LL= -2 Log Likelihood. Adjusted for sex, age, project, adopted status, race, and ethnicity, live together, educational attainment, and occupation. Bolded = *p* < .05.

1. IQ effect sizes were calculated according to the population expected standard deviation of 15 than the study sample of 11.9. [↑](#footnote-ref-1)
2. Analyses with qualitative measures were restricted to those that reported their specific hobbies, thus minimum reports represent those with at least 1 hobby that was at least somewhat cognitively demanding. [↑](#footnote-ref-2)
3. IQ effect sizes were calculated according to the population expected standard deviation of 15 than the study sample of 11.9. [↑](#footnote-ref-3)
4. Analyses with qualitative measures were restricted to those that reported their specific hobbies, thus minimum reports represent those with at least 1 hobby that was at least somewhat cognitively demanding. [↑](#footnote-ref-4)
